# Diet suppresses glioblastoma initiation in mice by maintaining quiescence of mutation-bearing neural stem cells

**DOI:** 10.1016/j.devcel.2023.03.021

**Published:** 2023-05-22

**Authors:** Valeria Amodeo, Timothy Davies, Amalia Martinez-Segura, Melanie P. Clements, Holly Simpson Ragdale, Andrew Bailey, Mariana Silva Dos Santos, James I. MacRae, Joao Mokochinski, Holger Kramer, Claudia Garcia-Diaz, Alex P. Gould, Samuel Marguerat, Simona Parrinello

**Affiliations:** 1 Samantha Dickson Brain Cancer Unit, UCL Cancer Institute, London WC1E 6DD, UK; 2 MRC London Institute of Medical Sciences, Du Cane Road, London W12 0NN, UK; 3 Institute of Clinical Sciences, Faculty of Medicine, Imperial College London, Du Cane Road, London W12 0NN, UK; 4 The Francis Crick Institute, 1 Midland Road, London NW1 1AA, UK

**Keywords:** neural stem cells, quiescence, tumour initiation, metabolism, fatty acid oxidation, p53

## Abstract

Glioblastoma is thought to originate from neural stem cells (NSCs) of the subventricular zone that acquire genetic alterations. In the adult brain, NSCs are largely quiescent, suggesting that deregulation of quiescence maintenance may be a prerequisite for tumor initiation. Although inactivation of the tumor suppressor p53 is a frequent event in gliomagenesis, whether or how it affects quiescent NSCs (qNSCs) remains unclear. Here, we show that p53 maintains quiescence by inducing fatty-acid oxidation (FAO) and that acute p53 deletion in qNSCs results in their premature activation to a proliferative state. Mechanistically, this occurs through direct transcriptional induction of PPARGC1a, which in turn activates PPARα to upregulate FAO genes. Dietary supplementation with fish oil containing omega-3 fatty acids, natural PPARα ligands, fully restores quiescence of p53-deficient NSCs and delays tumor initiation in a glioblastoma mouse model. Thus, diet can silence glioblastoma driver mutations, with important implications for cancer prevention.

## Highlights

• p53 maintains neural stem cell quiescence by inducing fatty-acid oxidation (FAO)
• p53 loss prematurely activates neural stem cells during gliomagenesis
• Restoration of FAO through diet reinstates quiescence to suppress tumor initiation

## Graphical abstract

**Figure:**
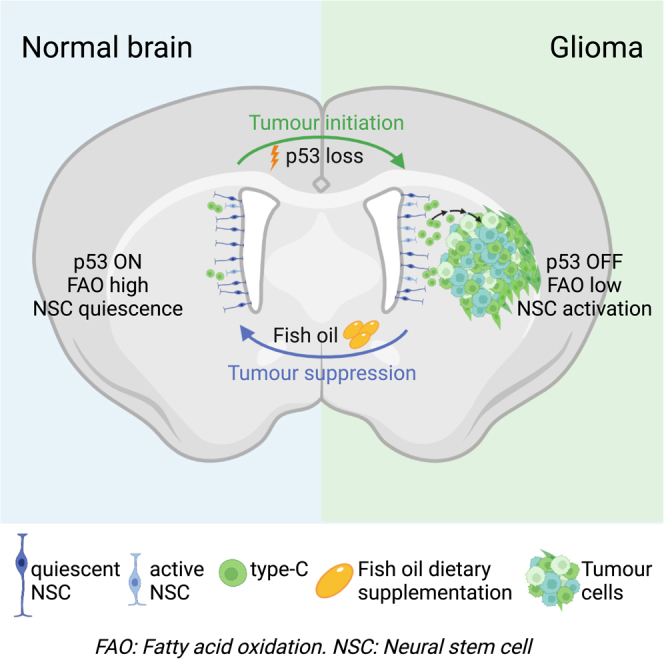

## Teaser

Amodeo et al. examine the impact of a glioma-relevant mutation on murine subventricular zone neural stem cells and identify premature quiescence exit driven by metabolic rewiring as a key tumor-initiating event. They show that a fish-oil-supplemented diet is sufficient to counteract these effects, thereby silencing mutations and suppressing tumorigenesis.

## Introduction

Increasing evidence indicates that glioblastoma (GBM) originates from neural stem cells (NSCs) of the subventricular zone (SVZ) neurogenic niche.^1^^,^^2^ SVZ NSCs, which are largely quiescent (qNSCs) in the normal adult brain,^1^ were shown to bear mutations in cancer-driving genes, including in *TERT*, *TP53* and *EGFR* in GBM.^2^ This suggests that these genes may control transitions between quiescence and activation to a proliferative state at the onset of tumorigenesis. Therefore, deciphering the impact of GBM-relevant mutations on the biology of qNSCs should provide understanding of disease etiology and reveal approaches for cancer prevention.

The transcription factor p53 (*TP53* in humans, *Trp53* in mice) is the most frequently mutated gene in human cancer, and the p53 pathway is altered in 87% of GBM patients.^3^ Interestingly, p53 has also been linked to the regulation of adult murine SVZ neurogenesis.^4^^,^^5^ In rodents, neurogenesis arises from a subpopulation of NSCs termed “type-B cells.”^6^ Although largely quiescent, adult murine type-B cells can activate to a proliferative state (active NSCs, [aNSCs]) from which they give rise to transit amplifying progenitors (type-C cells), which in turn fuel the production of neurons and glia.^6^ Analysis of constitutive p53 knockout mouse models showed that p53 restrains type-C cell proliferation and neuronal differentiation.^4^^,^^5^ In contrast, its role in qNSCs is less clear, with reports of no changes to a mild increase,^4^^,^^5^ likely due to compensatory effects. Here, we combined a conditional and inducible p53 knockout mouse model (*p53*^*icKO*^) with mechanistic assays and GBM mouse models to examine the function of p53 and its effectors in adult SVZ qNSCs and glioma initiation.

## Results

### p53 enforces NSC quiescence

**Figure 1:**
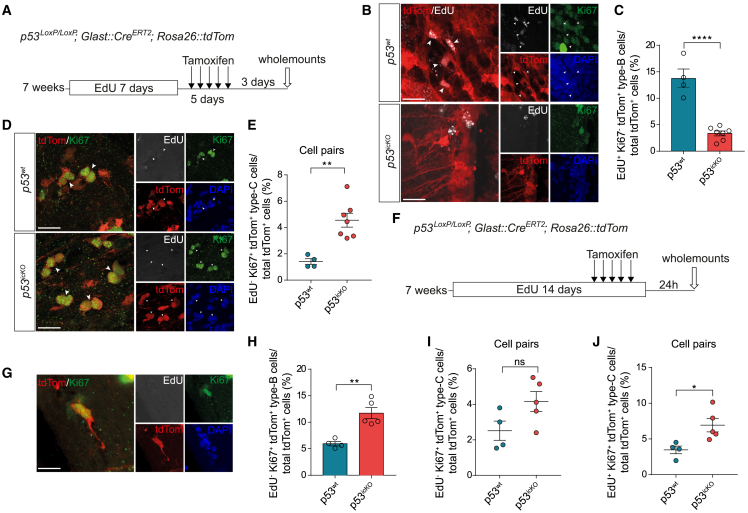
p53 controls NSC quiescence (A) Schematic of experimental outline. (B) Immunostaining of EdU^+^/Ki67^−^/tdTom^+^ label-retaining resting type-B cells (arrowheads) in *p53*^*wt*^ and *p53*^*icKO*^ SVZ whole mounts. (C) Quantification of resting type-B cells. *p53*^*wt*^ N= 4, *p53*^*icKO*^ N = 7. (D) Immunostaining of EdU^−^/Ki67^+^/tdTom^+^ pairs of type-C cells (arrowheads) in *p53*^*wt*^ and *p53*^*icKO*^ SVZ whole mounts. (E) Quantification of type-C cell pairs. *p53*^*wt*^, N = 4; *p53*^*icKO*^, N = 7. (F) Schematic of experimental outline. (G) Representative active type-B cell (aNSC). (H) Quantification of aNSCs. *p53*^*wt*^, N= 4; *p53*^*icKO*^, N = 5. (I and J) Quantification of EdU^−^/Ki67^+^/tdTom^+^ (I) and EdU^+^/Ki67^+^/tdTom^+^ (J) pairs of type-C cells. *p53*^*wt*^, N = 4; *p53*^*icKO*^, N= 5. All graphs represent mean ± SEM, unpaired, two-tailed Student’s t test. Scale bars: 20 μm. p values: ∗p < 0.05, ∗∗p < 0.01, ∗∗∗∗p < 0.0001. ns >0.05 See also Figure S1.

To examine the acute effects of p53 loss in qNSCs and lineage-trace recombined cells and their progeny, *p53*^*icKO*^ (*p53*^*LoxP/LoxP*^;*tdTom*^*fl/+*^;GLAST::CreERT2) or control mice (*p53*^*+/+*^;*tdTom*^*fl/+*^;GLAST::CreERT2) were treated with tamoxifen at 7 weeks of age.^4^^,^^5^ Following confirmation of recombination efficiency (>60%) (Figures S1A and S1B) and type-B cell specificity (Figures S1C and S1D) by immunocytochemistry and FACS analysis, respectively, we performed 5-ethynyl-2'-deoxyuridine (EdU)-label retention experiments to investigate effects of p53 loss both in type-B cells that had previously activated and returned to quiescence (resting qNSCs) and in type-B cells that had not yet activated (dormant qNSCs).^7^ For resting qNSCs, EdU was administered for 7 days, followed by a 5-day tamoxifen administration and analysis of the SVZ in whole-mount preparations 3 days later (Figure 1A). The percentage of resting qNSCs (identified as radial EdU^+^/Ki67^−^/tdTom^+^ cells) was markedly reduced in *p53*^*icKO*^ mice relative to controls, indicative of premature re-activation upon *p53* loss (Figures 1B and 1C). This was accompanied by an increase in pairs of EdU^−^/Ki67^+^ and Ascl1^+^/td-Tom^+^ type-C cells, which likely represent the immediate progeny of non-label-retaining recombined NSCs (Figures 1D, 1E, S1E, and S1F).^7^ In contrast, the numbers of 3- and 4-cell clusters were similar in control and *p53*^*icKO*^ animals (Figures S1G and S1H), confirming that effects were selective to type-B cells. To examine dormant qNSCs, we administered EdU for 14 days, a time-window during which most resting qNSCs incorporate EdU, while dormant qNSCs remain unlabeled.^7^ Recombination of p53 was induced during the last 5 days of the labeling period, and whole mounts analyzed 1 day later to identify dormant qNSCs undergoing activation based on Ki67 positivity and lack of EdU (Figure 1F). The loss of p53 induced premature activation of dormant qNSCs (Figures 1G and 1H), accompanied by a trend toward an increase in EdU^−^/Ki67^+^ type-C cell pairs, which did not reach significance (Figure 1I) and a significant increase in EdU^+^/Ki67^+^ type C cell pairs. The latter likely represented immediate progenitors of recombined qNSCs (resting and dormant) that activated during the tamoxifen/EdU administration period (Figure 1J). Thus, p53 maintains type-B cell quiescence and its deletion aberrantly activates resting and dormant qNSCs.

### p53 regulates fatty-acid oxidation in qNSCs

**Figure 2:**
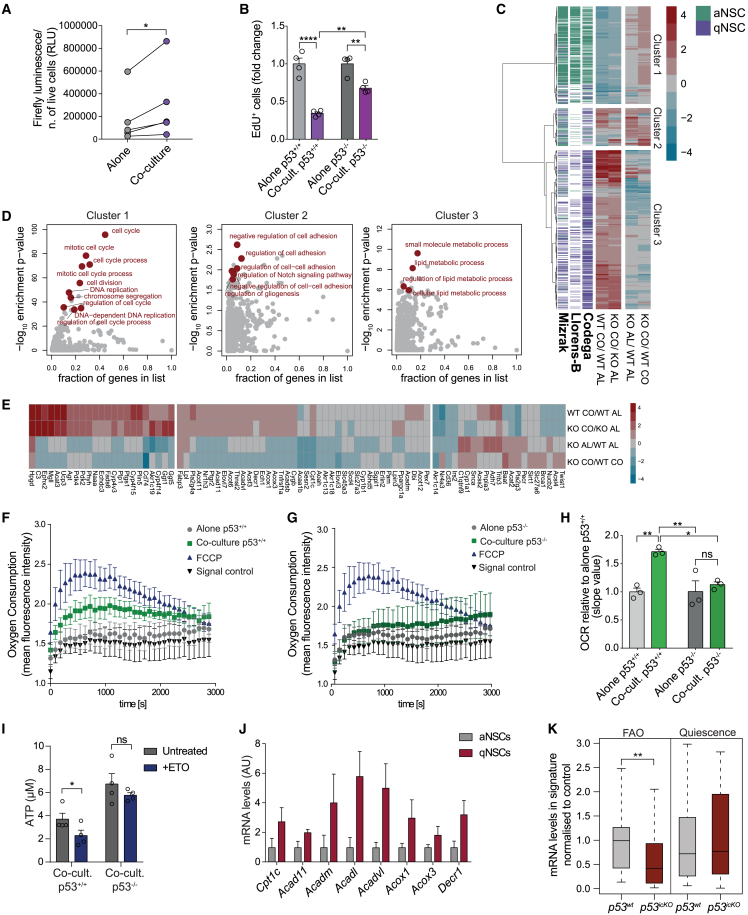
p53 regulates FAO in qNSCs (A) p53 promoter activity of indicated NPC cultures. n = 5. Paired two-tailed Student’s t test. (B) Quantification of EdU FACS profiles of *p53*^*+/+*^ and *p53*^*−/−*^ NPCs alone or co-cultured. Fold change. Mean ± SEM, n = 4. Two-way ANOVA with Tukey’s multiple-comparisons test. (C) Hierarchical clustering of RNA-seq log_2_ expression ratios between of *p53*^*+/+*^ (WT) and *p53*^*−/−*^ (KO) NPCs alone (AL) and co-cultured (CO) alongside indicated *in vivo* datasets.^9^^,^^10^^,^^11^ (D) GO term enrichment analysis of clusters from (C). (E) Hierarchical clustering of RNA-seq log_2_ expression ratios for lipid-metabolism genes. (F and G) FA-driven oxygen consumption of *p53*^*+/+*^ (F) and *p53*^*−/−*^(G) NPCs alone or co-cultured. Carbonyl cyanide 4-(trifluoromethoxy) phenylhydrazone (FCCP) is positive, background fluorescence is negative control. n = 3. (H) Quantification of oxygen consumption rate (OCR) in cultures from (F) and (G). Mean ± SEM, n = 3, two-way ANOVA with Tukey’s multiple-comparisons test. (I) Intracellular ATP in *p53*^*+/+*^ and *p53*^*−/−*^ NPCs co-cultures with or without etomoxir (ETO). Mean ± SEM, n = 4, two-way ANOVA with Sidak’s multiple-comparisons test. (J) qRT-PCR analysis of FAO genes in aNSCs and qNSCs FACS-purified from the SVZ of GFAP::GFP mice. Mean ± SEM, N = 3. (K) qRT-PCR analysis of FAO and quiescence genes in qNSCs FACS-purified from *p53*^*wt*^ and *p53*^*icKO*^ mice. N = 4; boxplots represent median and interquartile range. Two-sided Wilcoxon test. p values: ∗p < 0.05, ∗∗p < 0.01, ∗∗∗∗p < 0.0001. ns >0.05 See also Figure S2.

We next assessed mechanisms by which p53 enforces quiescence by exploiting an established co-culture assay in which neural stem/progenitor cells (NPCs) acquire phenotypic and molecular hallmarks of quiescence upon direct cell-cell contact with primary brain microvascular endothelial cells (bmvEC).^8^ To further validate this model, we transcriptionally profiled NPCs alone or in co-culture by RNA sequencing (RNA-seq) (Table S1). Comparison with published signatures of prospectively purified SVZ qNSCs,^9^^,^^10^^,^^11^ revealed that endothelial-induced quiescence mimics *in vivo* phenotypes to a significant extent, with an overlap of 384 upregulated (25.1% with *in vivo* qNSC signature, p = 3.7 × 10^−109^) and 246 downregulated genes (36.4% with *in vivo* aNSC signature, p = 1.1 × 10^−131^) (Figure S2A). Kyoto Encyclopedia of Genes and Genomes (KEGG) and Gene Ontology (GO) slim analysis showed recapitulation of quiescence-related processes, including upregulation of type-B fate markers, adhesion and signaling, lipid metabolism, and downregulation of cell proliferation (Figures S2B and S2C).^9^^,^^10^^,^^11^^,^^12^^,^^13^ Luciferase reporter assays confirmed that p53 activity increased in co-cultured NPCs compared with alone NPCs (Figure 2A). Furthermore, acute p53 recombination in primary *p53*^*LoxP/LoxP*^ NPCs (*p53*^*−/−*^ NPCs) resulted in a less pronounced cell-cycle arrest in co-culture relative to *p53*^*+/+*^ NPCs controls (Figures 2B, S2D, and S2E),^14^ confirming that the co-culture system reflects *in vivo* phenotypes and can inform p53 effectors.

We therefore examined p53-controlled transcriptional programs by subjecting *p53*^*+/+*^ and *p53*^*−/−*^ NPCs alone and in co-culture to bulk RNA-seq (Table S1). Hierarchical clustering of the genes shared between co-cultured NPCs and qNSCs *in vivo* (Figure S2A), identified three groups of genes in *p53*^*−/−*^ NPCs (Figures 2C and 2D; Table S1): genes that did not significantly change (cluster 2), increased (cluster 1) or decreased (cluster 3) in expression upon p53 loss. Cluster 2 comprised type-B cell identity genes, including glial markers, adhesion, and Notch signaling, suggesting that p53 does not control type-B fate.^9^^,^^10^ Consistently, immunostaining confirmed that GFAP expression and neurosphere-like morphology were similar between *p53*^*+/+*^ and *p53*^*−/−*^ NPC co-cultures (Figure S2E). Cluster 1 included cell-cycle genes, as expected^15^ (Figure 2B). Cluster 3 was enriched in lipid-metabolism signatures, suggesting that p53 may control qNSCs metabolic state.

**Figure 3:**
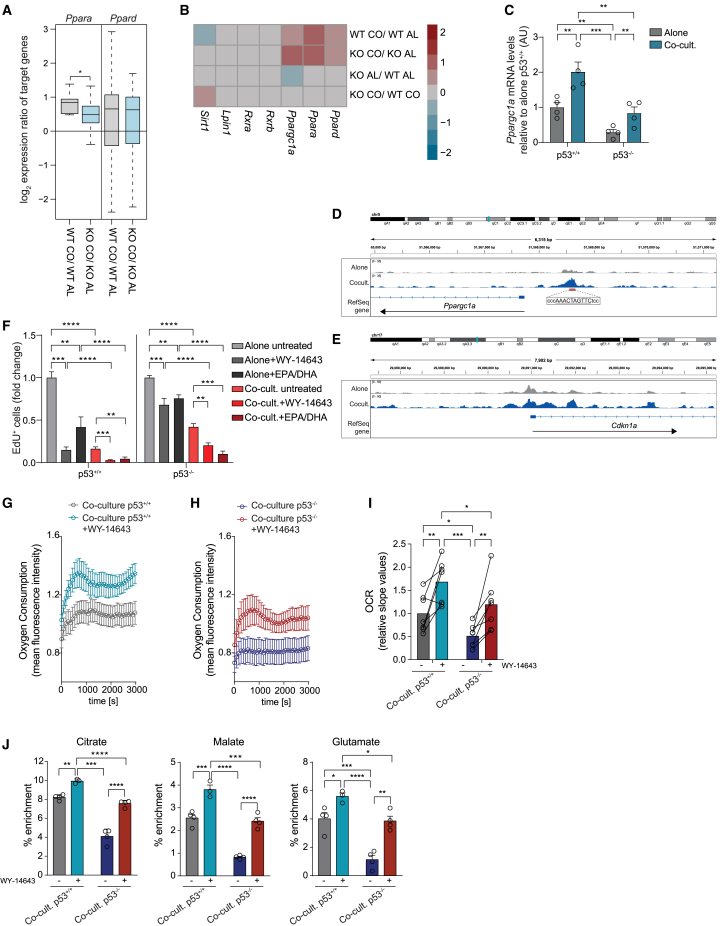
p53 mediates quiescence through PPARα (A) RNA-seq log_2_ expression ratios of *Ppara* and *Ppard* targets in indicated cultures. (B) RNA-seq log_2_ expression ratios of FAO regulators in indicated cultures. (C) qRT-PCR of *Ppargc1a* mRNA levels in *p53*^*+/+*^ and *p53*^*−/−*^NPCs alone and in co-culture. n = 4. (D and E) p53 CUT&RUN read coverage at *Ppargc1a* (D) and *Cdkn1a* (E) gene loci for alone and co-cultured NPCs. (F) Quantification of EdU FACS profiles of indicated cultures treated or untreated with PPARα agonist WY-14643 or omega-3 PUFAs (EPA/DHA). Fold change. n = 4. (G and H) FA-driven oxygen consumption in *p53*^*+/+*^ (G) and *p53*^*−/−*^ (H) NPC co-cultures treated or untreated with WY-14643. n = 7. (I) OCR of the signal profiles shown in (G) and (H). n = 7. (J) ^13^C enrichment in co-cultured *p53*^*+/+*^ and *p53*^*−/−*^ NPCs treated or untreated with WY-14643. n = 4. All graphs mean ± SEM except in (A) where boxplots represent median and interquartile range; two-sided Wilcoxon test (A), two-way ANOVA with Tukey’s multiple-comparisons test (C, F, I, and J). p values: ∗p < 0.05, ∗∗p < 0.01, ∗∗∗p < 0.001, ∗∗∗∗p < 0.0001. See also Figure S3.

In response to DNA damage, p53 mediates cell-cycle arrest predominantly through transcriptional activation of *p21*/*Cdkn1a*.^16^^,^^17^ We therefore assessed p21 function in our system, by comparing the response of wild-type (*p21*^*+/+*^) and p21 knockout (*p21*^*−/−*^) NPCs with co-cultured NPCs. Surprisingly, we found no differences (Figure S2F). Consistent with this, p53 binding to the *p21* promoter and *p21* mRNA levels were similar in alone and co-cultured wild-type NPCs (Figures 3E and S2G), indicating that p53-induced quiescence is p21 independent.

Fatty-acid oxidation (FAO) has emerged as an important mediator of stem cell quiescence.^18^^,^^19^^,^^20^^,^^21^ As *p53*^*−/−*^ NPC co-cultures displayed changes in lipid metabolism signatures (Figure 2D), we examined the role of FAO downstream of p53. Many enzymes involved in FA metabolism were deregulated in the transcriptomes of *p53*^*−/−*^ relative to *p53*^*+/+*^ NPCs (Figure 2E). To determine whether this was accompanied by functional changes, we first measured FA-driven oxygen consumption in *p53*^*+/+*^ and *p53*^*−/−*^ NPCs cultured alone and with bmvECs. While *p53*^*+/+*^ NPCs increased FAO in co-culture (Figures 2F and2H), *p53*^*−/−*^ NPCs did not (Figures 2G and 2H). Interestingly, despite FAO signatures being downregulated in both proliferating and quiescent *p53*^*−/−*^ NPCs, a change in metabolic state only occurred in co-culture, confirming the quiescence specificity of FAO.^18^ To determine whether p53-regulated FAO is required for energy production, we measured ATP and total adenine nucleotide pool levels in *p53*^*+/+*^ and *p53*^*−/−*^ co-cultures before and after treatment with the FAO inhibitor etomoxir (ETO). Both were significantly decreased in ETO-treated wild-type co-cultures, whereas no differences were detected in the absence of p53 (Figures 2I and S2H). This confirms that FAO is used for energy production^18^ and that p53 is a key regulator of this metabolic program.

Next, we assessed p53 regulation of FAO *in vivo*. First, we FACS-purified quiescent and active type-B cells from the SVZ of GFAP::GFP mice (identified as GFP^+^/CD24^−^/CD133^+^/EGFR^−^ and GFP^+^/CD24^−^/CD133^+^/EGFR^+^, respectively) and used quantitative real-time PCR to measure the expression of a panel of p53-regulated FAO genes (Figure 2E; Table S1). We found that expression levels were higher in qNSCs relative to aNSCs, consistent with increased lipid metabolism in quiescent cells (Figures 2J, S2I, and S2J).^9^^,^^10^ Furthermore, analysis of qNSCs FACS-purified from *p53*^*icKO*^ mice 24 h post-recombination (identified as tdTom^+^/CD24^−^/CD133^+^/EGFR^−^) (Figure S1C) revealed an overall downregulation of FAO genes, in the absence of changes in quiescence markers, confirming that p53 controls the FAO program (Figures 2K and S2K).

### p53 mediates quiescence through PPARα

**Figure 4:**
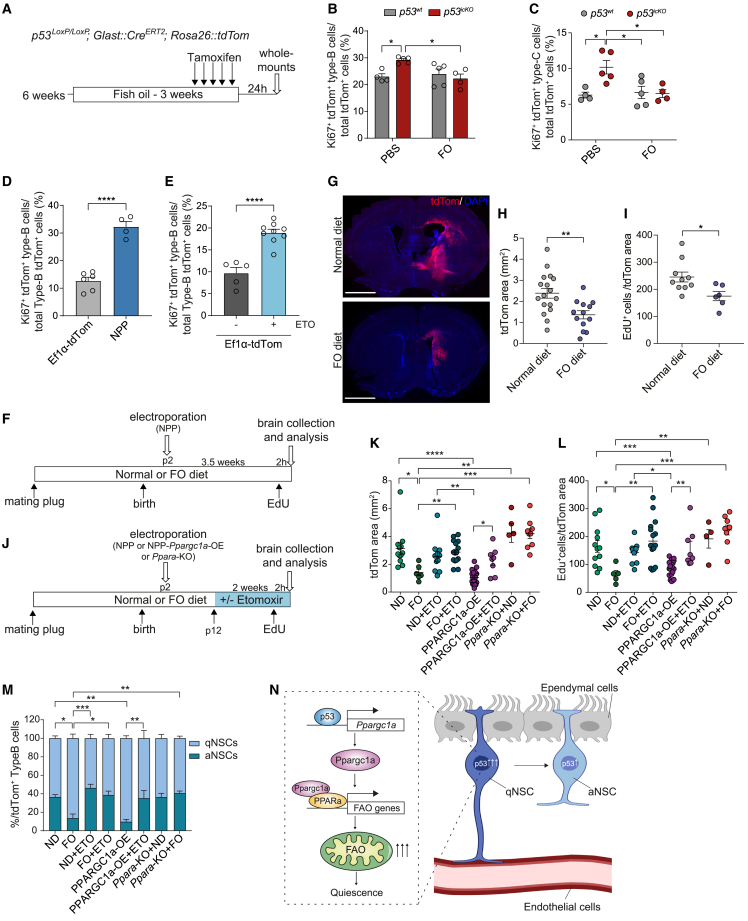
A fish-oil supplemented diet delays tumor initiation (A) Schematic of experimental outline. (B and C) Quantification of aNSCs (B) and proliferating type-C cell pairs (C) in *p53*^*w*t^ and *p53*^*icKO*^ mice administered PBS or fish oil (FO). p53^*w*t^ + PBS; N = 4; p53^icKO^ + PBS, N = 5; p53^*w*t^ + FO, N = 5; p53^icKO^ + FO, N = 4. (D) Quantification of aNSCs following electroporation of EF1α-tdTomato or NPP plasmids. EF1α-tdTom, N = 6; NPP, N = 4. (E) Quantification of aNSCs following electroporation of EF1α-tdTomato and treatment with etomoxir (+ETO, N = 9) or vehicle control (−ETO, N = 5). (F) Schematic of experimental outline. (G and H) Representative images (G) and quantifications (H) of tdTom-labeled tumors in mice fed normal (ND) or FO-supplemented diet. Scale bars: 2 mm. ND, N= 17; FO, N = 13. (I) Quantification of EdU^+^ cells normalized to tdTom^+^ tumor area. ND, N = 10; FO, N = 6. (J) Schematic of experimental outline. (K and L) Quantifications of tdTom area (K) and EdU^+^ cells (L) following electroporation with: NPP construct fed ND or FO diet with or without ETO; NPP constructs overexpressing *Ppargc1a* fed ND with (PPARGC1a-OE + ETO) or without (PPARGC1a-OE) ETO; NPP constructs expressing sgRNAs to *Ppara* fed ND (*Ppara*-KO + ND) or FO (*Ppara*-KO + FO). ND, N = 12; FO, N = 6; ND + ETO, N = 11; FO + ETO, N = 14; PPARGC1a-OE, N = 13; PPARGC1a-OE + ETO, N = 8; *Ppara*-KO + ND, N = 4; *Ppara*-KO + FO, N = 9. (M) Quantification of qNSCs and aNSCs in the SVZ of tumor-bearing mice in (K) and (L). (N) Model. All graphs represent mean ± SEM; two-way ANOVA with Tukey’s multiple-comparison test (B and C); unpaired two-tailed Student’s t test (D, E, H, and I); one-way ANOVA with Tukey’s multiple-comparisons test (K, L, and M). p values: ∗p < 0.05, ∗∗p < 0.01, ∗∗∗p < 0.001, ∗∗∗∗p < 0.0001. ns >0.05 See also Figure S4.

Our results suggest that p53 maintains qNSCs through transcriptional regulation of FAO genes. In qNSCs of the hippocampal neurogenic niche, FAO is partially controlled by peroxisome proliferator-activated receptor alpha (PPARα), a master regulator transcription factor of FAO genes.^18^ As most FAO genes dysregulated in *p53*^*−/−*^ NPCs (Figure 2E) are PPAR targets,^22^ we hypothesized that PPARs may also mediate p53 effects. Both *Ppara* and *Ppard* isoforms were expressed in NPCs and upregulated in co-culture (Figure 3B). However, while expression of target genes of both increased in quiescent NPCs, only *Ppara* targets were p53-dependent, suggestive of a p53/PPARα crosstalk (Figure 3A). To identify the underlying mechanisms, we examined expression of PPARs themselves, alongside their key regulators^23^ in the RNA-seq data and found PPARα coactivator peroxisome proliferator-activated receptor gamma coactivator 1-alpha (*Ppargc1a*/*Pgc1a*),^23^ to be strongly downregulated in *p53*^*−/−*^NPCs cultured alone (Figure 3B). Subsequent qPCR validation experiments revealed a parallel significant decrease in *Ppargc1* levels in *p53*^*−/−*^ relative to *p53*^*+/+*^NPCs co-cultures (Figure 3C). Furthermore, cleavage under targets and release using nuclease (CUT&RUN) analysis of wild-type NPCs, either alone or in co-culture, revealed direct binding of p53 at a response element within the *Ppargc1a* promoter, specifically upon quiescence (Figure 3D). Indeed, *Ppargc1a* was both bound by p53 in the CUT&RUN data and deregulated upon p53 loss in the RNA-seq data (Figure S3A). These results suggest a model whereby p53 activity in qNSCs induces *Ppargc1a* transcription. In turn, PPARGC1a enhances PPARα activity to induce transcription of lipid-catabolic enzymes resulting in increased FAO and quiescence (Figure 4N). To test this model functionally, we asked whether increasing PPARα activity through the administration of exogenous ligands would compensate for the PPARGC1a decrease in *p53*^*−/−*^ NSCs and rescue quiescence.^24^ Treatment with the PPARα agonist WY-14643 increased FAO genes expression and restored the cell-cycle arrest of *p53*^*−/−*^ co-cultured NPCs, without affecting stemness (Figures 3F and S3B–S3D). The WY-14643 rescue was dependent on PPARα and not caused by off-target effects because it was lost in *Ppara* knockout cells (Figures S3E, S4F, and S4G). To confirm that the effects of WY-14643 were mediated by FAO downstream of PPARα, we performed two parallel experiments. We measured the FA-driven oxygen consumption rate in quiescent *p53*^*+/+*^ and *p53*^*−/−*^ NPCs untreated or treated with WY-14643 and found a complete rescue of FAO in *p53*^*−/−*^ NPCs upon WY-14643 treatment (Figures 3G–3I). Next, we exposed quiescent *p53*^*+/+*^ and *p53*^*−/−*^ NPCs to ^13^C-palmitate in the presence or absence of WY-14643 and traced the incorporation of radiolabeled carbons into tricarboxylic acid (TCA) cycle intermediates and amino acids derived from TCA intermediates using gas chromatography-mass spectrometry (GC-MS). We found that while ^13^C incorporation into both was significantly decreased in untreated *p53*^*−/−*^ co-cultures, as expected, WY-14643 treatment restored it to the levels of *p53*^*+/+*^ co-cultures (Figures 3J and S3F). WY-14643 treatment of *p53*^*+/+*^ NPCs monocultures also phenocopied the cell-cycle arrest of *p53*^*+/+*^ NPC co-cultures while treatment of *p53*^*+/+*^ NPC co-cultures resulted in a more pronounced arrest, which was paralleled by an increase in FAO in both metabolic assays, further confirming the link between fatty-acid metabolism and quiescence (Figures 3F and 3G). These experiments indicate that p53 mediates quiescence at least in part through regulation of FAO via a PPARGC1a/PPARα axis.

### Dietary fish-oil supplementation delays tumor initiation

Our results suggest that premature activation of qNSCs through metabolic remodeling may be a mechanism by which p53 mutations drive GBM. We therefore hypothesized that restoring PPARα-dependent FAO downstream of p53 loss may suppress tumorigenesis. PPARα is a nutrient sensor and can be activated by dietary polyunsaturated FAs (PUFAs), providing a potential strategy for tumor prevention through diet.^25^ To test this, we first examined *in vitro* effects of docosahexaenoic acid (DHA) and eicosapentaenoic acid (EPA) omega-3 PUFAs, the main components of fish oil. Treatment of *p53*^*+/+*^ and *p53*^−/−^ NPC co-cultures with EPA and DHA increased expression levels of FAO genes in both genotypes, restoring them to wild-type levels in *p53*^−/−^ NPCs (Figure S3G). This was accompanied by a PPARα-dependent rescue of quiescence in *p53*^−/−^ NPCs (Figures 3F and S3E) and further suppression of proliferation in *p53*^*+/+*^ cells both alone and in co-culture as observed with WY-14643 (Figure 3F). Next, we tested the *in vivo* effects of dietary supplementation with fish oil. Fish oil, or PBS control, were administered to *p53*^*wt*^ or *p53*^*icKO*^ mice for a total of 3 weeks, and during the last 5 days tamoxifen was added prior to analysis of the SVZ 24 h later (Figure 4A). Strikingly, fish-oil supplementation fully reversed the effect of acute p53 loss in qNSCs *p53*^*icKO*^, suppressing the increase in activated type-B and early type-C progenitors (Figures 4B and 4C).

To determine whether diet could impact oncogenic transformation, we examined the role of the identified p53/PPARGC1a/PPARα/FAO pathway in tumor initiation from NSCs using a somatic mouse model of GBM based on CRISPR-Cas9 gene editing and PiggyBac transposition technology.^26^^,^^27^^,^^28^ The model is driven by the combined inactivation of the tumor suppressor genes *Nf1, Pten*, and *Trp53* (alongside tdTomato overexpression, hereafter NPP model) in endogenous postnatal SVZ NSCs via electroporation (Figure S4B). As in genetically engineered mouse models carrying the same mutations,^29^^,^^30^ the NPP model gives rise to brain tumors with histological and molecular features of GBM.^27^^,^^28^ Importantly, the introduction of the mutations dramatically increased qNSC activation, relative to control mice electroporated with a control tdTomato plasmid (Figures 4D and S4A), indicating that aberrant activation of qNSCs is an early event in tumor initiation. Furthermore, acute treatment of tdTomato-electroporated brains with an 8-day course of ETO during the same time period (p4–p11), phenocopied effects of p53 deletion in qNSC leading to their increased activation to a proliferative state compared with controls (Figures 4E and S4A). To determine whether p53 phenotypes could be reversed by diet in this tumor model, females were fed a high-fish-oil or normal control diet from conception through lactation. Tumorigenesis was then induced in the pups and brain tissue analyzed 3.5 weeks later, a time at which early lesions can be detected in control animals (Figures 4F and 4G). Remarkably, resulting lesions were significantly smaller and less proliferative in the high-fish-oil diet-fed group relative to controls, as judged by tdTomato^+^ area and EdU incorporation, respectively (Figures 4G–4I). To determine whether fish-oil administration acts through FAO, we repeated the protocol described above in the presence or absence of a 14-day course of ETO (Figure 4J) and found that FAO inhibition completely abrogated fish-oil effects, leading to the development of larger and more proliferative lesions, similar to early NPP control tumors (Figures 4K and 4L). Furthermore, we assessed whether FAO induction downstream of p53 and fish oil depends on the identified PPARGC1a/PPARα axis. The NPP construct was modified to incorporate the *Ppargc1a* gene (Figure S4B), resulting in its overexpression in the tumor cells (Figures S4C and S4E) and, importantly, upregulation of p53-regulated FAO genes (Figure S4D). *Ppargc1a*-overexpressing NPP tumors induced in animals fed a normal diet were significantly smaller and less proliferative than controls, phenocopying fish-oil effects (Figures 4K and 4L). Furthermore, *Ppargc1a* tumor-suppressive effects were fully reversed by ETO administration, confirming that they were FAO-dependent (Figures 4K and 4L). We next deleted the *Ppara* gene by introduction of a CRISPR sgRNA to *Ppara* into the NPP construct (Figure S4B, S4F and S4G) and used this construct to initiate tumors in animals fed a control or high-fish-oil diet. Remarkably, *Ppara* deletion abrogated the effects of fish oil, leading to the development of tumors of similar size as control NPP tumors fed a normal diet. In normal diet, NPP tumor development was unaffected by knockout of *Ppara*, as expected for p53-deficient tumors with compromised *Ppara* activity (Figures 4K and 4L). Thus, fish oil suppresses initiation through PPARα-mediated FAO.

Finally, to confirm that these effects resulted from dysregulation of NSC quiescence, we assessed the proportions of qNSCs and aNSCs in all experimental groups described above (Figure 4M). Tumor size was directly related to NSC activation state, with all groups in which p53/PPARGC1a/PPARα/FAO was disrupted displaying aberrant activation; those in which the pathway was rescued, namely fish-oil administration and *Ppargc1a* overexpression, retaining a much greater proportion of qNSCs in the SVZ. These experiments further underscore the functional link between FAO and quiescence. We conclude that diet counteracts tumor-initiating mutations to delay gliomagenesis.

## Discussion

Mounting evidence suggests that normal tissues commonly bear a variety of genetic changes, including driver mutations.^31^^,^^32^^,^^33^ How these mutations remain silent despite their tumor-initiating potential, is a fundamental and still unresolved question in cancer biology. It has been proposed that a normal tissue microenvironment acts as a tumor-suppressive mechanism by dominantly keeping mutations in check.^31^^,^^34^ Our study identifies diet as a key contributing factor in mutation silencing.

Mutations in p53 that have been found to be shared between tumor-free SVZ and matching tumors of GBM patients^2^ pointing to NSCs as GBM cells of origin and to p53 as a key tumor-initiating mutation. We found that p53 loss contributes to tumor initiation by prematurely activating qNSCs, which is consistent with observations in other niches.^35^^,^^36^^,^^37^^,^^38^^,^^39^ Surprisingly, this was independent of its canonical effector p21, as it occurred through regulation of FAO, highlighting the dominant role of the cellular metabolic state in directing decisions between quiescence and activation.^18^^,^^19^^,^^20^^,^^21^

Unlike in other systems where p53 directly controls FAO genes,^40^^,^^41^ in qNSCs p53 induces FAO via a PPARGC1a/PPARα axis. There is a precedent for a bi-directional crosstalk between p53 and PPARGC1a and a role for the latter in controlling FAO in cancer.^42^^,^^43^^,^^44^^,^^45^ It is tempting to speculate that the p53/PPARGC1a/PPARα may represent a general homeostatic pathway in normal cells, which is highjacked in cancer to promote tumor growth by rewiring the cell metabolic state.

These findings have important therapeutic implications as we showed that, in the context of the NPP model, early GBM development was dramatically suppressed by fish oil. This suggests that dietary intervention may be an effective therapeutic strategy for suppressing tumor initiation. As quiescent stem cells often share a common metabolic profile and can act as cancer cell-of-origin across many tissues, dietary intervention may provide a more general approach for cancer prevention.^46^

### Limitations of the study

It is possible that additional effectors besides increased FAO mediate quiescence downstream of p53 and future studies should explore this possibility. It would be equally important to examine the long-term impact of fish-oil supplementation on long-term survival and the relevance of our mouse findings to human disease.

## STAR★Methods

### Key resources table

**Table:** 

REAGENT or RESOURCE	SOURCE	IDENTIFIER
**Antibodies**		

Rabbit monoclonal anti-Ki67	Abcam	Cat# ab16667; RRID: AB_302459
Mouse monoclonal anti-Ascl1 (Clone 24B72D11.1)	kindly gifted by F. Guillemot (Francis Crick Institute, UK). Urbán et al.^47^	N/A
Mouse monoclonal anti-p53	Cell Signaling	Cat# 2524; RRID_331743
Rabbit polyclonal anti-GFAP	Agilent	Cat# Z0344; RRID: AB_10013382
Mouse monoclonal anti-Sox2	Abcam	Cat# ab79351;RRID: AB_10710406
Mouse monoclonal anti-Nestin	Santa Cruz	Cat# sc-33677; RRID: AB_627995
Monoclonal anti-mCD24 PE (Clone M1/69)	BD Pharmingen	Cat# 12-0242-82; RRID: AB_465602
Monoclonal anti-mCD133 Biotin (Clone 13A4)	eBioscience	Cat# 13-1331-82; RRID: AB_466591
PE-Cy7 conjugated streptavidin	eBioscience	Cat# 25-4317-82; RRID: AB_10116480
Rabbit polyclonal anti-RFP	Antibodies Online	Cat# ABIN129578 RRID: AB_10781500
Monoclonal anti-mCD24 eFluor (Clone M1/69)	eBioscience	Cat# 48-0242-82; RRID: AB_1311169
Monoclonal anti-PPAR alpha	Arigo Biolaboratories	Cat# ARG55240; N/A

**Bacterial and Virus Strains**		

Ad-CMV-iCre	Vector Biolabs	Cat# 1045
Ad-CMV-Null	Vector Biolabs	Cat# 1300

**Chemicals, Peptides, and Recombinant Proteins**		

EGF-complexed to Alexa Fluor™ 647	ThermoFisher	Cat# E35351
Tamoxifen	Sigma	Cat# T5648
EdU (5-ethynyl-2′-deoxyuridine)	Santa Cruz	Cat# sc-284628
*Fish oil*	Sigma	Cat# F8020
PPARa agonist WY-14643	Sigma	Cat# C7081
Etomoxir	Sigma	Cat# E1905
Palmitic acid	Sigma	Cat# P5585
BSA Conjugated Docosahexaenoic Acid (DHA, 22:6n-3)	Cloud-Clone Corp	Cat# CPO122Ge11
BSA-conjugated Eicosapentaenoic Acid (EPA, 20:5n-3)	Cloud-Clone Corp	Cat# CPO632Ge11

**Critical Commercial Assays**		

Zombie Green™ Fixable Viability Kit	BioLegend	Cat# 423111
Click-iT™ EdU Alexa Fluor™ 647 Imaging kit	Life Technologies	Cat# C10340
Click-iT™ EdU Alexa Fluor™ 647 Flow Cytometry assay	Life Technologies	Cat# C10419
Fatty Acid Oxidation Assay kit	Abcam	Cat# ab217602
Extracellular Oxygen Consumption Assay kit	Abcam	Cat# ab197243
iScript gDNA clear cDNA synthesis kit	Bio-rad	Cat# 1725034

**Deposited Data**		

RNA-seq data	This paper	GEO: GSE165801
CUT&RUN data	This paper	GEO: GSE165802
Codega et al RNA-seq dataset	Codega et al.^9^	GEO: GSE54653
Llorens-Bobadilla et al RNA-seq dataset	Llorens-Bobadilla et al.^10^	GEO: GSE67833
Mizrak et al RNA-seq dataset	Mizrak et al.^11^	GEO: GSE109447

**Experimental Models: Cell Lines**		

Mouse: Primary mouse NPCs isolated at P9–P12	This paper	N/A
Mouse: Primary mouse brain microvascular endothelial cells	Cell Biologics	Cat# C57-6023

**Experimental Models: Organisms/Strains**		

Mouse: FVB/N-Tg(GFAPGFP)14Mes/J	The Jackson Laboratory	Cat# Jax003257; RRID: IMSR_JAX:003257
Mouse: B6.Cg-*Gt(ROSA)26Sor*^*tm14(CAG-tdTomato)Hze*^/J	The Jackson Laboratory	Cat# 007914 RRID: IMSR_JAX:007914
Mouse: *p53*^*LoxP/LoxP*^	Marino et al.^48^	N/A
Mouse: GLAST::CreERT	Mori et al.^49^	N/A
Mouse: C57Bl/6J	Charles River	RRID: IMSR_JAX:000664

**Oligonucleotides**		

	See Table S2 for primer sequences	N/A

**Software and Algorithms**		

Fiji ImageJ	Schindelin et al.^50^	https://imagej.net/Fiji
FlowJo version 10.6.2	Becton, Dickinson and Company	https://www.flowjo.com/
TopHat v.2.0.11	Kim et al.^51^	https://ccb.jhu.edu/software/tophat/index.shtml
DESeq2 Bioconductor package	Love et al.^52^	http://bioconductor.org/packages/release/bioc/html/DESeq2.html
HTSeq v.0.6.1	Anders et al.^53^	http://htseq.readthedocs.io/en/release_0.9.1/overview.html
Trimmomatic v.036	Bolger et al.^54^	http://www.usadellab.org/cms/index.php?page=trimmomatic
Bowtie2 v2.3.4.3	Langmead and Salzberg^55^	http://bowtie-bio.sourceforge.net/bowtie2/index.shtml
Chippeakanno R package v3.20.1	Zhu et al.^56^	https://www.bioconductor.org/packages/release/bioc/html/ChIPpeakAnno.html
VLAD	Richardson and Bult^57^	http://proto.informatics.jax.org/prototypes/vlad/
MACS2 v2.1.1	Zhang et al.^58^	https://hbctraining.github.io/Intro-to-ChIPseq/lessons/05_peak_calling_macs.html
g:Profiler package	Raudvere et al.^59^	https://biit.cs.ut.ee/gprofiler/gost

### Resource Availability

#### Lead Contact

Further information and requests for resources and reagents should be directed to and will be fulfilled by the Lead Contact, Simona Parrinello (s.parrinello@ucl.ac.uk).

#### Materials Availability

This study did not generate new unique reagents.

### Experimental Model and Subject Details

#### Mice

All procedures were performed in compliance with the Animal Scientific Procedures Act, 1986 and approved by the UCL Animal Welfare and Ethical Review Body (AWERB) in accordance with the International guidelines of the Home Office (UK). GFAP::GFP mice were obtained from The Jackson Laboratory (Jax 003257). *p53*^*icKO*^ were generated by crossing GLAST::CreERT2 mice to animals carrying a loxP-flanked *Trp53* gene (*p53*^*LoxP/LoxP*^) or *p53*^*+/+*^ and to a Rosa26::tdTom inducible reporter strain (Jax 007914).^48^^,^^49^^,^^60^ Animals were in a mixed 129xC57BL/6J background and both males and females were analysed between 7-10 weeks of age. For tumour initiation experiments, C57Bl6/J mice were purchased from Charles River (Jax 000664). Approximately an equal number of male and female mice were used per experiment. Mice were group-housed (where possible) in individually ventilated cages and maintained with 12-hour light/dark cycles with water and chow available *ad libitum*.

#### Neural progenitor cell culture

NPCs were isolated from the SVZ of postnatal day 9-12 (P9–P12) mouse brains as previously described.^8^ Briefly, following microdissection, the SVZ was digested by incubation in HBSS (Invitrogen, 14170-088) supplemented with 0.25% trypsin and 60 U ml^−1^ Dnase I (Sigma, D4263) for 2 minutes at 37°C. Single cell suspensions were plated onto poly-L-lysine (PLL)-coated plates in SVZ explant medium consisting of DMEM/F12 (Invitrogen, 11320074), 3% FBS (Invitrogen), 20 ng ml^−1^ EGF (Peprotech, 315-09-1000) for 48 h. NPCs were routinely grown, for up to 8–10 passages, in SVZ culture medium consisting of DMEM/F12 (Life technologies, 11320074), 0.25% FBS, N2 (Life technologies, 17502001), 20 ng ml^−1^ EGF, 10 ng ml^−1^ bFGF (Peprotech, 450-33A) and 35 μg ml^−1^ bovine pituitary extract. For the *Cdkn1a* loss-of-function experiments, NPCs were isolated from the SVZs of *p21*^*+/+*^ or *p21*^*-/-*^ mice, a kind gift of Dr Owen J. Sansom^61^ (Cancer Research UK Beatson Institute, Glasgow, UK).

#### Brain microvascular endothelial cell culture

C57BL/6J primary mouse brain microvascular endothelial cells (bmvEC) were purchased from Cell Biologics (C57-6023) and subcultured on plates coated with attachment factor protein (Life Techonologies, S006110) in Endothelial Cell Growth Medium 2 (Promocell, C-22111).

### Method details

#### Tamoxifen, EdU, Fish oil and Etomoxir administration

Tamoxifen (Sigma, T5648) was administered to 7-9 weeks-old mice by intraperitoneal injection (i.p.) at 100mg/kg/d. For assessment of resting type-B cells, EdU (Santa Cruz, sc-284628) was administered in the drinking water (0.2 mg/mL) *ad libitum* for 7 days, followed by tamoxifen administration for five consecutive days and mice were sacrificed 3 days later. This protocol enables identification of resting qNSCs by labelling type-B cells that incorporated EdU during the 7-day pulse and then re-entered quiescence during the 8-day chase period, thereby appearing as EdU^+^/Ki67^-^/tdTom^+^ cells with radial morphology. To examine dormant type-B cells, EdU was administered in the drinking water *ad libitum* for 14 days. Mice were injected with tamoxifen over the last 5 days of EdU labelling and sacrificed 24h later. This enables identification of dormant qNSCs that activate during the 1-day chase period as radial EdU^-^/Ki67^+^/tdTom^+^ cells. For Omega-3 FA administration experiments, 6-weeks old mice were given daily 100μL of fish oil (Sigma, F8020) corresponding to 0.2-0.43 g/kg/d docosahexaenoic acid (DHA) and 0.4-0.67 g/kg/d eicosapentaenoic acid (EPA) by oral gavage for 3 weeks. Tamoxifen was administered over the last 5 days of fish oil administration and SVZs collected 24h later. To prevent oxidation of the fish oil, aliquots were protected from direct light and supplemented with 40μM EDTA and 0.5mg/ml Ascorbyl Palmitate (Sigma, PHR1455). Control 6-weeks old mice received 100μL of PBS containing 40μM EDTA and 0.5mg/ml Ascorbyl Palmitate. For the tumour initiation experiments, we used a *de novo* somatic GBM model based on CRISPR/Cas9-mediated deletion of *Nf1*, *Pten* and *Trp53* tumour suppressors (NPP), as described.^27^ Briefly, sgRNAs (single guide RNA) were expressed from a PiggyBac vector alongside a tdTomato reporter to fluorescently label resulting tumours. PiggyBase transposase was co-expressed with Cas9 in a second non-integrating plasmid. NPP*-Pparpgc1a-OE* and NPP*-Ppara-*KO constructs were generated using InFusion Kit (Clontech, 638917) and T4 DNA Ligase (NEB, M0202S), following manufacturer’s instructions. To generate the NPP*-Ppara-*KO, sgRNA to target *Ppara* (5’-GCCGGGGGACTCGTCCGTGC-3’), as reported,^62^ was cloned in the NPP plasmid 3’ of the *Nf1, Pten, Trp53* sgRNAs. Mouse *Ppargc1a* CDS was cloned after the tdTomato sequence as a polycistronic construct with a T2A linker for the generation of the NPP*-Pparpgc1a*-OE plasmid.^27^^,^^28^ C57BL/6J female mice were randomly placed on an *ad libitum* fish oil supplemented diet (Teklad Global 2020X diet supplemented with 30-32g fish oil/kg containing 13.5% EPA and 10.5% DHA) or control diet (Teklad Global 2020X diet) following the observation of a copulation plug. Pups from these females were injected at P2 with the two plasmids mixed at a ratio of 0.8:1 using a Femtojet microinjector (Eppendorf) directly into the lateral ventricle. Plasmids were electroporated into the sub-ventricular zone using the Gemini X2 Generator set for 5 square pulses, 50 msec/pulse at 100 volts, with 950 msec intervals^63^. Mice were maintained on fish oil supplemented diet or control diet until collection. After 3.5 weeks animals were i.p. injected with EdU (50mg/kg) 2 hours prior to sacrifice by transcardial perfusion of paraformaldehyde (PFA, 4%) under terminal anaesthesia. The brains were collected, stored overnight in PFA at 4°C prior to Vibratome sectioning (50μm) and subsequent analysis of tumour development. For the inhibition of FAO during tumour initiation *in vivo*, pups were treated with etomoxir (Sigma-E1905) starting 10 days after electroporation and continuing for 2 weeks until sacrifice. This was achieved through lactation via etomoxir i.p. injections of dams at 10mg/kg every other day during the first week and through i.p. etomoxir injection of the pups at 5mg/kg every other day during the second week. For the analysis of acute NSC activation in response to tumour-initiating mutations, pups at P2 were electroporated with the EF1α-tdTomato (control) or NPP (*Nf1, Pten, Trp53*) plasmids and SVZ wholemount preparations were analysed 9 days after electroporation (P11). To assess effects of FAO inhibition of NSC activation, EF1α-tdTomato pups received either PBS (vehicle) or etomoxir at P4 through lactation via i.p. injection to the dams every other day (10 mg/kg) and collected at P11.

#### FACS

qNSCs and aNSCs from the SVZ were FACS purified from GFAP::GFP mice as previously described^9^ using the following antibodies: anti-mCD24 PE (1:2000; BD Pharmingen 12-0242-82), biotin conjugated anti-mCD133 (1:100, eBioscience 13-1331-82) and PE-Cy7 conjugated streptavidin (1:1000, eBioscience 25-4317-82) and EGF-complexed to Alexa Fluor™ 647 (1:100, ThermoFisher E35351). For isolation of *p53*^*w*t^ and *p53*^*icKO*^ qNSCs, mice were sacrificed 24h after tamoxifen administration. Upon dissociation, cells were stained with anti-mCD24-eFluor450 (1:2000; eBioscience 48-0242-82), biotin conjugated CD133 (1:100) and PE-Cy7 conjugated streptavidin (1:1000) and EGF-complexed to Alexa Fluor™ 647 (1:100). Zombie Green™ Fixable Viability Kit (1:1000, Biolegend 423111) or DAPI (1:10000, Sigma D9542) were used to assess cell viability. For both sets of experiments cell populations were manually gated based on FMOs, sorted using FACS Aria III (BD Biosciences) and collected into RLT buffer for RNA extraction.

#### Co-culture experiments and cell treatments

Co-culture experiments were performed as previously described and analysed 48h later.^8^ In brief, bmvEC were plated at a density of 2.6x10^4^cells/cm^2^. The following day, NPCs were plated either alone or onto endothelial monolayers at a density of 34x10^3^cells/cm^2^. After 48h, the NPCs were removed by selective trypsinization before analysis. Treatments were as follows: PPARα agonist WY-14643 was purchased from Sigma (C7081) and added to NPCs alone or in co-culture with bmvECs at the concentration of 200μM for 24h. BSA-conjugated Eicosapentaenoic Acid (EPA, 20:5n-3) (Cloud-Clone Corp., #CPO122Ge11) and BSA Conjugated Docosahexaenoic Acid (DHA, 22:6n-3) (Cloud-Clone Corp., #CPO632Ge11) were used at the concentration of 50μM each for 6h or 24h. For the EdU incorporation assay, 10μM EdU was added to the culture media for 2h prior to fixation and detection using the Click-iT™ EdU Alexa Fluor™ 647 Flow Cytometry Assay Kit (Thermo Fisher Scientific, C10424), according to manufacturer’s instructions. DNA was stained with DAPI. The cells were analysed on Fortessa X20s flow cytometer (BD). For *p53* loss-of-function experiments, *p53*^*loxP/loxP*^ NPCs were infected with adenoviruses expressing a codon-improved Cre (iCre) under a CMV promoter (Ad-CMV-iCre, Vector Biolabs, #1045). Adenoviruses carrying CMV promoter only were used as controls (Ad-CMV-Null, Vector Biolabs, #1300). NPCs were used within 2-3 passages post infection to minimise compensatory effects of p53 loss. For luciferase assays, NPCs were nucleofected with a reporter plasmid containing the p53 binding element (PG13-CAT) prior to seeding in alone and bmvEC co-culture for 48h.^64^^,^^65^ Luminescence was normalized to viable cell numbers in each condition. For the analysis of adenine nucleotides, NPCs were treated with etomoxir at the final concentration of 100μM starting on day 2 of co-culture for 24h.

#### Immunohistochemistry and Immunofluorescence

SVZ wholemounts were prepared as previously described.^66^ For Ascl-1 staining, mice were perfused with normal saline prior to dissection to reduce background from endogenous IgG in the blood. SVZs were fixed overnight in 4% PFA at 4°C, permeabilised for 1.5h in blocking solution, consisting of PBS with 10% donkey serum and 2% Triton X-100 at RT and incubated for 48h in primary and secondary antibodies diluted in PBS containing 2% TritonX-100 and 10% donkey serum at 4°C. Primary antibodies were: rabbit anti-Ki67 (1:50, abcam ab16667) and mouse anti-Ascl1 (1:200, a kind gift from Francois Guillemot55 (Francis Crick Institute, London, UK)). EdU was detected using Click-iT® EdU Imaging Kit (ThermoFisher Scientific, C10340). For immunofluorescence experiments, cells were fixed in 4% PFA, permeabilised in 0.5% Triton X-100, blocked in PBS containing 10% serum for 1h and incubated in primary antibody diluted in blocking buffer O/N at 4°C. Primary antibodies were: mouse anti-p53 (1:500, Cell Signaling 2524), rabbit anti-GFAP (1:1000, Dako Z0344), mouse anti-SOX2 (1:100, abcam ab79351), mouse anti-NESTIN (1:200, santa cruz sc- 33677), mouse anti-PPARα (1:25, Arigo Biolabs ARG55240). Alexa Fluor conjugated secondary antibodies (ThermoFisher Scientific) were diluted in 10% serum in DAPI (1:10000 in PBS) and incubated at RT for 1h. CellTracker™ Green (ThermoFisher Scientific, C7025) was used to label NPCs prior to seeding. Imaging was carried out using the Zeiss LSM880 confocal microscope. TdTomato targeted neural stem cells in brain coronal sections were stained anti-RFP (1:2000, Antibodies Online ABIN129578). Quantifications were performed by using Fiji ImageJ.^50^ For all wholemount quantifications, percentages of type-B and type-C were calculated over the total number of tdTom^+^ type-B and type-C cells.

#### FAO assay

Fatty acid oxidation was measured using the commercially available Fatty Acid Oxidation (Abcam, ab217602) and Extracellular Oxygen Consumption Assay kits (Abcam, ab197243) according to the manufacturer’s instructions. Treatment with 25μM FCCP served as positive and samples without cells as negative controls, respectively. Extracellular O_2_ consumption was measured every 90 seconds for 2h at Ex/Em=380/650 nm using the VarioSkan LUX Plate reader set at 37°C. (ThermoFisher Scientific). Oxygen Consumption Rate (OCR) was determined by calculating the slope of the linear proportion of the signal profiles.

#### Sample extraction for LC-HRMS analysis

Cell samples were extracted with 500 μL of extraction solvent (water/methanol, 20:80 v/v) and they were spiked with a known concentration of AMP 13C1015N5, ADP 15N5 and ATP 13C10 for their calibration curves (standard addition). Cell extracts were sonicated in an ultrasonic water bath (15 min) and centrifuged (13,000 g, 10 min, 5°C). Pooled quality controls (QCs) were created by pooling equal aliquots of each study sample, in order to assess technical reproducibility across the batch.

#### Nucleotides analysis by LC-HRMS

Liquid chromatographic analyses were performed on a Vanquish Flex Binary UHPLC system (Thermo Scientific Inc., MA, USA) coupled to a benchtop hybrid quadrupole-Orbitrap Q Exactive mass spectrometer (Thermo Scientific Inc., Bremen, Germany). Chromatographic separation of extracts was achieved using a SeQuant® ZIC®-cHILIC column (3μm,100Å 100 x 2.1 mm) held at a temperature of 45°C and a flow rate of 0.30 mL/min. Mobile phase was consisted of 90% acetonitrile with 10 mM ammonium acetate pH = 4.6 (solvent A) and 10 mM ammonium acetate pH = 4.6 (solvent B). The gradient elution started with 5% of solvent B increasing to 45% of B over 14 min. This condition was kept constant until 15.5 min followed by re-equilibration at the initial conditions, yielding a total run time of 20 min. Ionization was performed in the negative mode using a heated electrospray ionisation source, with the following parameters: spray voltage 3.0 KV, heater temperature 400°C, capillary temperature 320°C, S-lens RF level 50, sheath and auxiliary gas flow rate, 48 and 11 units, respectively. The mass accuracy was calibrated before sample analysis. Mass spectrometric data were acquired at high-resolution (70,000 at m/z 200) in profile mode using a Full MS scan method (m/z 70 to 700). Automatic gain control (AGC) was set to 1e6 and maximum injection time 250 ms. Xcalibur version 4.1 was used for data acquisition and processing. Peak areas of the Extracted Ion Chromatograms (EIC) were used in the data processing.

#### ^13^C-palmitate tracer experiments and GC-MS analysis

A 100mM stock of [U-^13^C]-palmitic acid (Sigma, 605573) was prepared in ethanol. This was diluted 100-fold into DMEM/F12 media containing 2% w/v fatty acid-free BSA (Sigma A8806) and sonicated for 15 minutes in a water bath sonicator at room temperature to form soluble BSA/palmitate complexes. The resulting media contained 1mM [U-^13^C]-palmitic acid, 2% BSA and 1% ethanol (170 mM) with a palmitate:BSA molar ratio of 3.3:1. Following filtration through a 0.2 μm PES filter (Millipore, Burlington, MA) this was diluted 1:10 in SVZ culture medium, giving a final concentration of 100μM [U-^13^C]-palmitic acid.

Cells in co-culture, in the presence or absence of PPARα agonist WY-14643 (200μM), were incubated with 100μM U-^13^C-palmitate SVZ medium for 6 hr or 24 hr. Cell were selectively dissociated from the endothelial monolayer and rapidly quenched on dry ice/ethanol. Samples were then pelleted at 0°C, washed with ice-cold PBS pH 7.4, transferred to 2 mL tubes and metabolites extracted at 4°C for 1 hr (with 3 x 8 min sonications in a water bath sonicator) using 600 mL chloroform/methanol (2:1, v/v, containing ^13^C lauric acid internal standard at 5nmol per sample). Extracts were transferred to 1.5 mL tubes and dried in a SpeedVac. The pellet was then re-extracted with 450 mL methanol/water (2:1, v/v, containing 1 nmol scyllo-Inositol internal standard; 4°C, 8 min sonication), the extract was combined with the first extract and then re-dried. Polar and apolar metabolites were separated by phase partitioning with chloroform/methanol/water (1:3:3, v/v/v) and then analyzed by GC-MS.

GC-MS data acquisition was performed largely as previously described,^67^ using an Agilent 7890B-5977A GC-MSD in EI mode after derivatization of twice methanol-washed dried extracts by the addition (a) for polar metabolites of 20 μL of a 20 mg/mL solution of methoxyamine hydrochloride in pyridine (both Sigma) at room temperature for >16 hr and 20 μL BSTFA + 1% TMCS (Sigma) at RT for >1 hr, or (b) for fatty acids of 25 μL chloroform/methanol (2:1, v/v) and 5 μL MethPrepII (Grace Alltech) at room temperature with no incubation. GC-MS parameters were as follows: carrier gas, helium; flow rate, 0.9 mL/min; column, DB-5MS (Agilent); for polar analyses: inlet, 270°C; temperature gradient, 70°C (2 min), ramp to 295°C (12.5°C/min), ramp to 320°C (25°C/min, 3 min hold); for apolar analyses: inlet, 250°C; temperature gradient, 70°C (1 min), ramp to 230°C (15°C/min, 2 min hold), ramp to 325°C (25°C/min, 3 min hold). Scan range was m/z 50-550 (polar) and 50-565 (apolar). Data were acquired using MassHunter software (version B.07.02.1938). Data analysis was performed using MANIC software, an in house-developed adaptation of the GAVIN package.^68^ Metabolites were identified and quantified by comparison to authentic standards, and label incorporation estimated as the percentage of the metabolite pool containing one or more ^13^C atoms after correction for natural abundance.

#### Quantitative RT-PCR

For *in vitro* experiments, RNA was extracted using RNeasy mini kit (Qiagen, 74104) following the manufacturer’s instructions. RNA was reverse transcribed using iScript gDNA clear cDNA synthesis kit (Bio-rad, 1725034) and quantitative PCR was performed using the qPCRBIO SyGreen Mix Lo-Rox (PCR Biosystems, PB20.11). For assessment of acutely FACS-purified qNSCs and aNSCs, RNA was extracted using RNeasy Plus MicroKit (Qiagen, 74034) according to the manufacturer’s instructions and cDNA libraries were prepared using the Smart-seq2 protocol.^69^ Primers used are listed in Table S2.

#### RNA-Sequencing

For RNA sequencing, RNA was isolated using RNeasy mini kit (Qiagen, 74104) according to manufacturer’s instructions. Libraries were prepared using the Truseq mRNA stranded kit and quality control checks were performed by Qubit and Bioanalyser analysis. Libraries were then pooled and run on a MiSeq Nano Flow Cell (V2 reagents) (Single Read 26 cycles) to check the balance of the libraries within the pool and adjusted where necessary. Clonal clusters of each library were then amplified onto an Illumina Flow Cell using the Illumina cBot system and sequenced on a HiSeq 2500 (v4 chemistry) as a Paired-End 100bp.

#### RNA-seq data pre-processing and differential expression analysis

Raw reads were aligned to the mouse genome (NCBI Build 37, USCS mm9) using the TopHat v.2.0.11 software^51^ and assigned to genomic features using HTSeq v.0.6.1.^53^ Differential expression analysis was performed and normalized counts were generated using the DESeq2 Bioconductor package^52^ (Table S1).

#### CUT&RUN library preparation and sequencing

CUT&RUN experiments were performed as previously described.^70^ In brief, 5x10^5^ NPCs grown either in alone or in coculture conditions were attached to concanavalin A–coated magnetic beads (Bangs Laboratories, BP531) and incubated overnight at 4°C in 0.05% Digitonin-Antibody buffer containing a rabbit anti mouse-p53 Ab (1:100 Leica, NCL-p53-CM5p). Samples incubated with rabbit IgG (1:150) were used as control. After incubation, samples were washed in Dig-wash and incubated with ProteinA-MNase fusion protein at 700 ng/mL (EpiCypher, 15-1016-EPC) on a tube rotator at 4 °C for 1 hr. Chromatin was digested in Incubation Buffer containing 1M CaCl_2_ at 0 °C for 10 minutes, chromatin fragments were released by incubation at 37 °C for 30 min. Chromatin was purified by performing phenol chloroform extraction. CUT&RUN barcoded libraries for Illumina sequencing were prepared using the NEBNext® Ultra™ II DNA Library Prep Kit for Illumina® (NEB, E7645) accordingly with Zhu et al.^71^ PCR amplification products were cleaned and size selected by using AMPure XP beads (Beckman Coulter, A63881). The resulting purified libraries were quantified by Qubit. Library size distribution was checked by using Agilent Bioanalyzer® High Sensitivity DNA chip (Agilent Technologies, 5067-4626). Indexed libraries were pooled and Illumina Paired-End (42x42bp, 6-bp index) sequencing was performed using Nextseq 500 platform with NextSeq 500/550 High Output Kit v2 (75 cycles).

#### CUT&RUN data pre-processing and peak calling

Sequencing data was analysed using the CUT&RUNTools pipeline.^71^ Briefly, Fastq files were trimmed using Trimmomatic^54^ before alignment to Mus musculus GRCm38 (mm10) genome with Bowtie2 (using the --dovetail option).^55^ Aligned reads were converted to bed format using bedtools bamtobed with -bedpe option, before filtering for fragments shorter than 120bp. Normalisation of each sample to mm10 read depth was performed using bedtools genomecov with a scale factor generated by division of an arbitrary large number by read depth for each sample. Peaks were called using MACS2.^58^ Downstream peak analysis was performed in R. Peaks were annotated using the Chippeakanno R package^56^ and visualised in IGV.

#### Bioinformatics analysis

RNA-seq and CUT&RUN functional analysis was performed using custom R scripts. Hierarchical clustering was performed using the “pheatmap” package. Figure 2C: Genes with an absolute DEseq2 log_2_ ratio > 1 in one or more contrast and defined as aNSCs or qNSCs markers in one the three published studies were used.^9^^,^^10^^,^^11^ Figure 2D: GO term enrichment analysis of Figure 2C clusters was performed using VLAD (http://proto.informatics.jax.org/prototypes/vlad/)^57^ Figure 2E: Genes with an absolute DEseq2 log_2_ ratio > 0.5 and adjusted p-value < 0.5 in the KO AL/WT AL or KO CO/WT CO contrasts and belonging to the fatty acid metabolic process GO category (GO_0006631) were used. Figure S2A: Genes with an absolute DEseq2 log_2_ ratio > 1 in the WT CO/WT AL contrast were used. Figures S2B and S2C: Genes with an absolute DEseq2 log_2_ ratio > 1 (up) or < -1 (down) and adjusted p-value < 0.05 in the WT CO/WT AL contrast were used for functional analysis using the g:Profiler package (Figure S2B, https://biit.cs.ut.ee/gprofiler/gost),^59^ or VLAD (Figure S2C, http://proto.informatics.jax.org/prototypes/vlad/).^57^ Figure 3A: Genes significantly regulated (DESeq2 adjusted p-value < 0.05) in the WT CO/WT AL contrast and found to be targets of Ppara but not Ppard or Pparg, or targets of Ppard but not Ppara or Pparg were used.^22^ Figure 3B: Expression ratios for 7 marker genes in indicated conditions. Figure S3A: Genes significantly regulated (DESeq2 adjusted p-value < 0.05) in either the KO AL/WT AL or KO CO/WT CO contrasts and showing significant p53 binding in at least one of four CUT&RUN experiments (MACS Q value < 0.01) were used.^58^

### Quantification and statistical analysis

Statistical analysis was performed using GraphPad7 or GraphPad9 built in tools. All graphs represent the mean±SEM unless otherwise indicated. Significance is stated as follows: p>0.05 (ns), p<0.05 (^∗^), p<0.01 (^∗∗^), p<0.001 (^∗∗∗^), p<0.0001 (^∗∗∗∗^). Two-tailed Student’s t test was used for statistical comparisons between two groups. Ordinary one-way ANOVA and two-way ANOVA with Tukey’s multiple comparisons or with Sidak’s multiple comparisons were used to determine statistical significance of multiple comparisons. Statistical details of experiments can be found in the figure legends. All experiments for which quantifications were performed were carried out a minimum of three times as indicated in the figure legends.

## Data Availability

• The scRNA-seq and CUT&RUN data have been deposited at GEO. Accession numbers are listed in the key resources table. • This paper does not report original code. • Any additional information required to reanalyse the data reported in this paper is available from the lead contact upon request.

## Supplemental information

Document S1. Figures S1–S4, Table S2, and supplemental references

Table S1. DEseq2 analysis, normalized and raw RNA-seq counts reported in this study, related to Figures 2C, 2D, and S2A

Document S2. Article plus supplemental information
