# Supplementary material for: Diet suppresses glioblastoma initiation in mice by maintaining quiescence of mutation-bearing neural stem cells
Source: Dev Cell. 2023 May 22;58(10):836–846.e6. doi: 10.1016/j.devcel.2023.03.021 (PMC10618406; doi:10.1016/j.devcel.2023.03.021)
Supplement: Document S1. Figures S1–S4, Table S2, and supplemental references [file mmc1.pdf]

Developmental Cell, Volume *58*

Supplemental information

Diet suppresses glioblastoma initiation in mice by maintaining quiescence of mutation-bearing neural stem cells

Valeria Amodeo, Timothy Davies, Amalia Martinez-Segura, Melanie P. Clements, Holly Simpson Ragdale, Andrew Bailey, Mariana Silva Dos Santos, James I. MacRae, Joao Mokochinski, Holger Kramer, Claudia Garcia-Diaz, Alex P. Gould, Samuel Marguerat, and Simona Parrinello

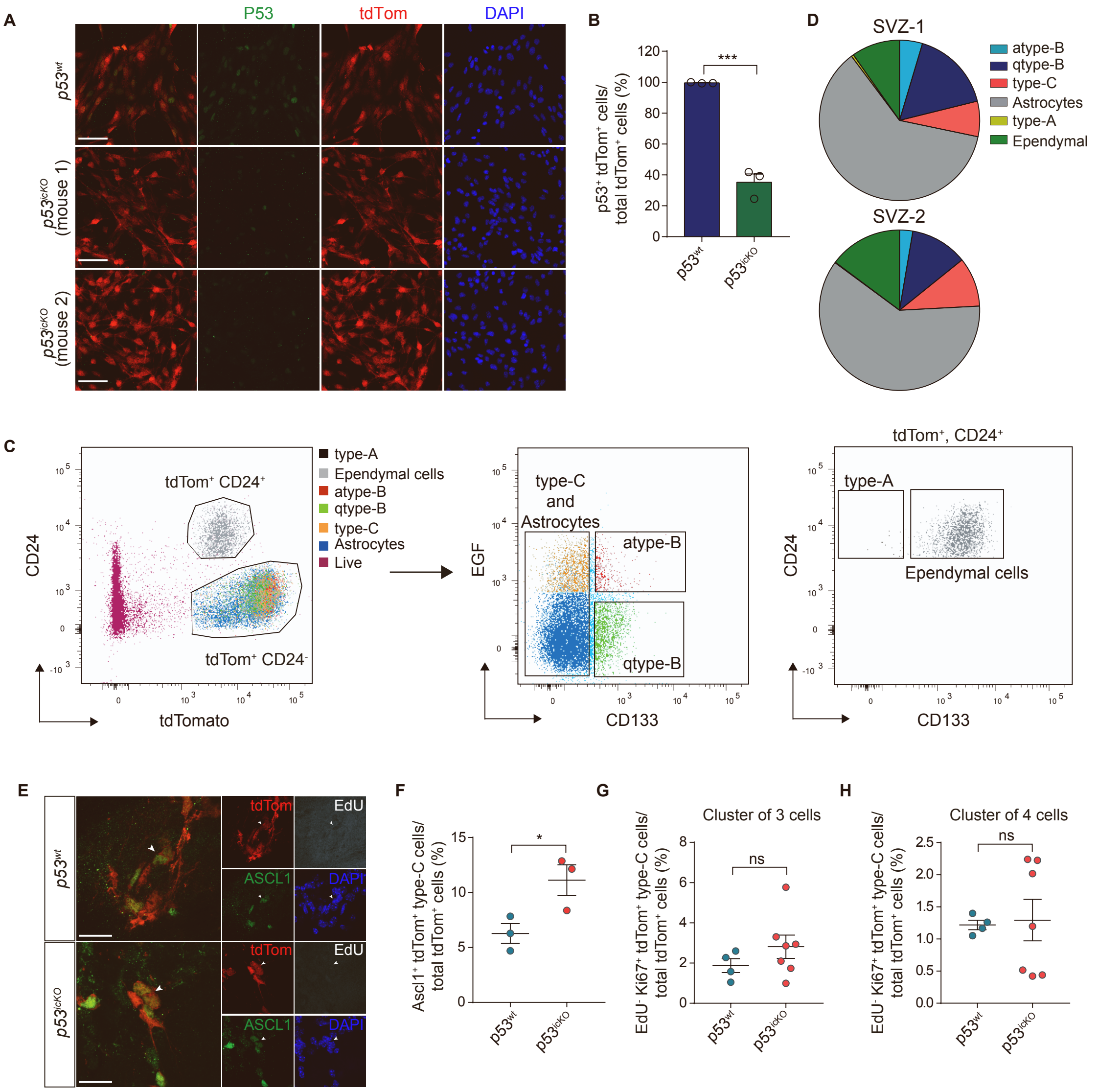

Figure S1. Selectivity of the *p53*^{icKO} model, Related to Figure 1.

A, Representative fluorescence images showing p53 expression in NPCs isolated from the SVZ of adult mouse brain 24h after tamoxifen administration. p53 is shown in green, recombined NPCs are tdTom$^+$ and nuclei are counterstained with DAPI (blue). Scale bar=100µm. **B,** Quantification of the percentage of p53$^+$ NPCs over the total number tdTom$^+$ NPCs. Mean±SEM, *p53*^{wt} n=3, *p53*^{icKO} n=3. ***p<0.001, unpaired two-tailed Student t-test. **C,** Representative FACS plots showing the gating strategy for all tdTom$^+$ SVZ subpopulations in *p53*^{icKO} mice. tdTom$^+$/CD24$^-$ cells were selected and gated on EGF-AF647 and CD133-PE-Cy7 levels to define tdTom$^+$/CD24$^-$/EGFR$^+$/CD133$^+$ aNSC cells, tdTom$^+$/CD24$^-$/EGFR$^-$/CD133$^+$ qNSCs and tdTom$^+$/CD24$^-$/EGFR$^-$/CD133$^-$ and tdTom$^+$/CD24$^-$/EGFR$^+$/CD133$^-$ astrocytes. Proportion of type-C cells was estimated from the tdTom$^+$/CD24$^-$/EGFR^{high}/CD133$^-$ population and distinguished from astrocytes based on cell size. Ependymal cells were defined from the tdTom$^+$/CD24$^+$ population as tdTom$^+$/CD24$^+$/CD133$^+$ cells and type-A as tdTom$^+$/CD24$^+$/CD133$^-$ cells. Cell viability was assessed with Viability Dye Zombie Green. Single immuno-stained and Fluorescence Minus One (FMO) SVZ cell suspensions were used as controls. tdTom$^-$ cell suspension served as negative control. **D,** Pie chart showing percentages of each subpopulation in *p53*^{wt} 24h post-recombination (see schematic in Figure 1A). **E,** Representative fluorescence images of Ascl1$^+$/tdTom$^+$ type-C cells in SVZ wholemounts of *p53*^{wt} and *p53*^{icKO} mouse brains 3 days post-recombination. White arrowheads denote pairs of Ascl1$^+$/tdTom$^+$ type-C cells. Scale bar=20µm. **F,** Quantification of the percentage of

Ascl1^{+}/tdTom^{+} type-C cell pairs over the total number of tdTom^{+} type-B and type-C cells. Mean±SEM, *p53^{wt}* n=3, *p53^{icKO}* n=3. *p<0.05, unpaired two-tailed Student t-test. **G-H,** Quantification of the percentage of EdU^{-}/Ki67^{+}/tdTom^{+} clusters of 3 type-C cells (G) and 4 type-C cells (H) in SVZ wholemounts from *p53^{wt}* and *p53^{icKO}* mice subjected to experimental protocol described in Fig.1A for the detection of resting qNSCs. Mean±SEM, *p53^{wt}* n=4, *p53^{icKO}* n=7. ns=not significant, unpaired two-tailed Student t-test.

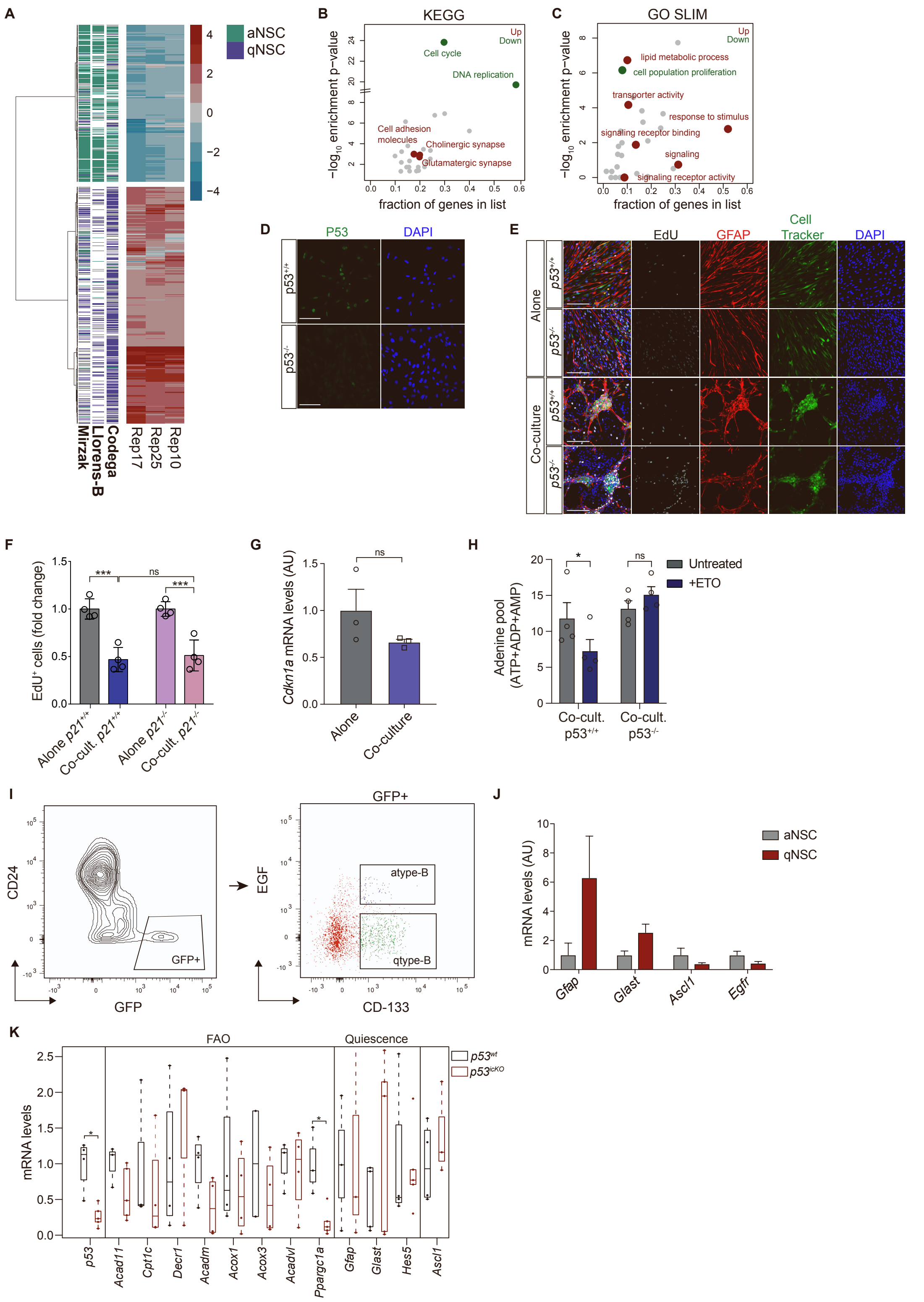

Figure S2. p53-mediated cell cycle arrest is p21-independent, Related to Figure 2.
A, Hierarchical clustering of RNA-seq log_2 expression ratios for three independent NPC co-culture experiments (Rep10, Rep17 and Rep25) relative to alone controls shown alongside published data-sets[S1,S2,S3] from type-B cells FACS acutely purified from the V-SVZ. **B-C,** KEGG and GO pathway analysis of genes up- and down-regulated upon co-culture. Enrichment p-values are shown as a function of the fraction of regulated genes present in KEGG pathways (B) and GO categories (C). Selected terms enriched in genes up- (red) and down-regulated (green) upon co-culture are highlighted. **D,** Representative immunofluorescence p53 staining

(green) in NPCs infected with an adenovirus expressing Cre recombinase (*p53*$^{-/-}$) or a control adenovirus (*p53*$^{+/+}$). DAPI-stained nuclei are in blue. Scale bar=100µm. **E,** Representative EdU staining of *p53*$^{+/+}$ and *p53*$^{-/-}$ NPCs cultured alone or with bmvECs for 48h and pulsed with EdU for 2h prior to fixation. NPCs are cell-tracker labelled (green) and stained for GFAP (red), EdU (grey) and DAPI (blue). Scale bar=20µm. **F,** EdU FACS profiles of *p21*$^{+/+}$ and *p21*$^{-/-}$ NPCs cultured alone or with endothelial cells for 48h and pulsed with EdU for 2h. Fold changes normalized to the respective alone controls are shown for each genotype. Mean±SEM, n=4 independent experiments. ns=not significant, ∗∗∗p<0.001, Two-way ANOVA with Tukey's multiple comparisons test. **G,** Quantitative RT-PCR analysis of *Cdkn1a* mRNA levels in NPCs alone and in co-culture. Fold changes relative to alone control are shown. Mean±SEM. n = 3, ns=not significant, unpaired two-tailed Student t-test. **H**, Adenine pool size (∑ATP+ADP+AMP) in *p53*$^{+/+}$ and *p53*$^{-/-}$ NSC in co-culture treated with etomoxir (ETO) for 24h. Untreated co-cultured cells were used as control. Mean±SEM, n=4 independent replicates, *p<0.05, Two-way ANOVA with Sidak's multiple comparisons test. **I**, Representative FACS plots of the strategy used to prospectively purify SVZ subpopulations using GFAP::GFP mice as in Codega et al[S1]. Cell viability was assessed with DAPI. Single immuno-stained SVZ cell suspensions from wild-type mice were used to set FACS gates. **J,** Quantitative RT-qPCR analysis of the mRNA levels of *Gfap*, *Glast*, *Ascl1* and *Egfr* in qNSCs and aNSCs cells FACS-purified as in **I** confirms successful enrichment of each subpopulation. Data represent the average of 3 independent sorts; Mean±SEM. **K**, qRT–PCR analysis of FAO and quiescence genes in qNSCs acutely FACS-purified from the SVZ of *p53*^{wt} and *p53*^{icKO} mice 24h following tamoxifen administration shown in Figure 2K as signature. Boxplots represent median, interquartile range, and most extreme data points that are not more than 1.5 times the interquartile range. n=4 independent sorts. For *p53* p=0.032, for *Ppargc1a* p=0.036, two-sided Wilcoxon test.

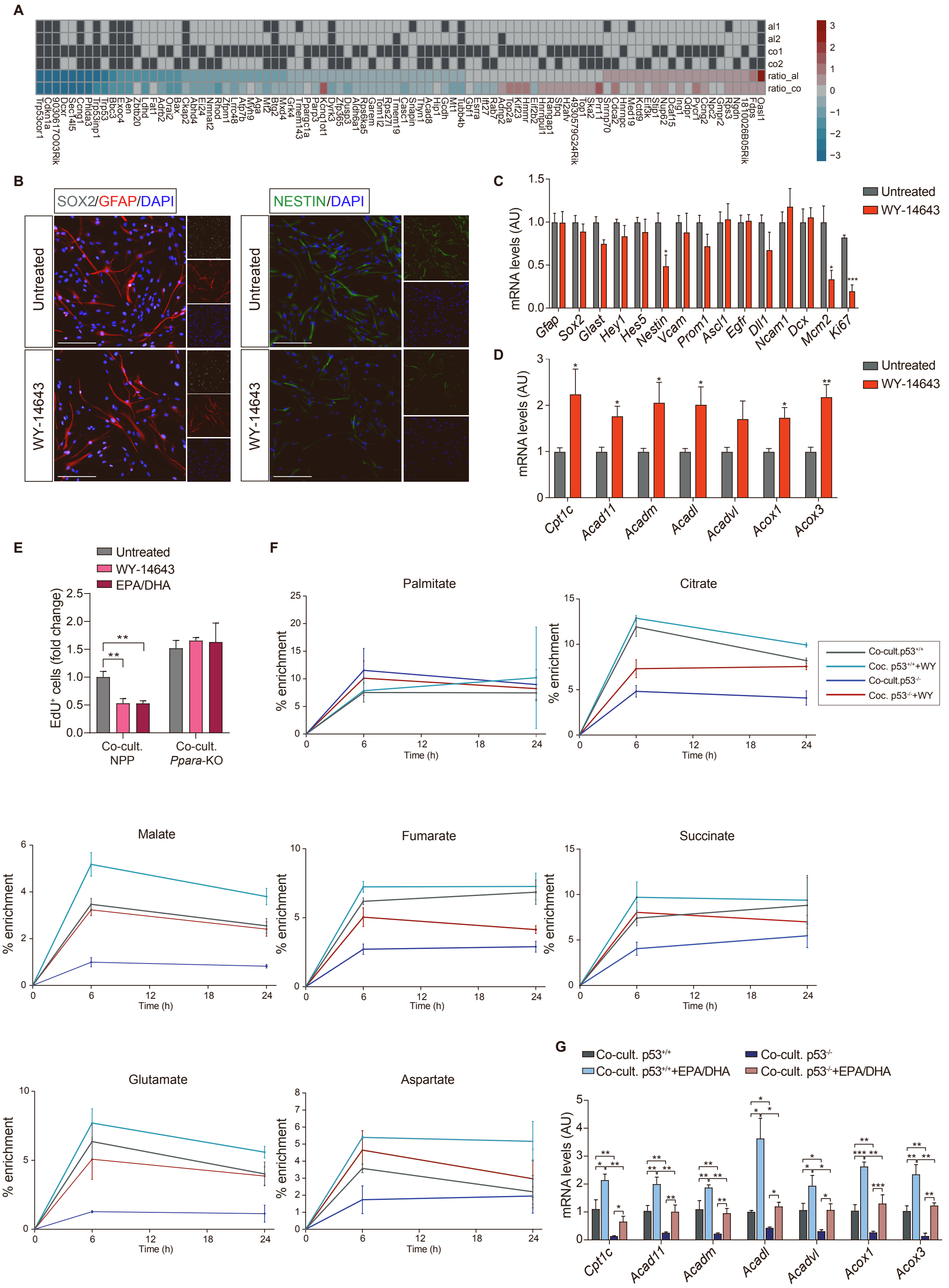

Figure S3. Validation of CUT&RUN experiments and WY-14643 and EPA/DHA treatments, Related to Figure 3.

A, List of genes regulated upon p53 deletion in alone or co-culture conditions, which show significant binding by p53 in CUT&RUN analysis. Rows 1-4: alone (al) and co-culture (co) CUT&RUN experiments for two independent replicates are shown and dark grey rectangle represents significant p53 binding. Rows 5-6: RNA-seq log_2 expression ratios between and *p53*$^{-/-}$ and *p53*$^{+/+}$ NPCs in alone (ratio_al) and in co-culture (ratio_co). **B,** Representative fluorescence images of SOX2 (grey), GFAP (red) and NESTIN (green) in NPCs untreated or treated with WY-14643 for 24h. Nuclei are counterstained with DAPI (blue). Scale bar=100µm. Note that PPARα agonist treatment does not induce NPC differentiation. **C-D,** Quantitative RT–

PCR analysis of markers for qNSC (*Gfap*, *Sox2*, *Glast*, *Hey1*, *Hes5*, *Nestin, Vcam, Prom1*), aNSC/TAPs (*Ascl1, Egfr, Dll1, Ncam1, Dcx*), proliferation (*Mcm2 and Ki67*) (C) and FAO genes (*Cpt1c*, *Acad11*, *Acadm*, *Acadl*, *Acadvl*, *Acox1* and *Acox3*) (D) in NPCs after 24h of WY-14643 treatment. Data are shown as fold change relative to untreated NPCs (control). n=5 independent experiments, Mean±SEM, *p<0.05, **p<0.01, ***p<0.001, unpaired two-tailed Student t-test. **E**, FACS quantification of EdU^+ NPP and NPP-*Ppara*-KO cells co-cultured with bmvEC for 24 h in the presence or absence of WY-14643 or EPA/DHA. Mean±SEM, **p<0.01, n=3 replicates, Two-way ANOVA with Sidak's multiple comparisons test. **F**, ^{13}C enrichment following 6h or 24h labelling with [U-^{13}C]-palmitic acid. Near isotopic steady-state enrichments are observed for palmitate, tricarboxylic acid (TCA) cycle intermediates and the amino acids glutamate and aspartate. $p53^{+/+}$ and $p53^{-/-}$ NPCs in co-culture untreated and treated with the PPARα agonist WY-14643 for 24h were used. Graphs shows the % of ^{13}C enrichment for all isotopologues of each metabolite at 6h and 24h. **G**, Quantitative RT–PCR analysis of FAO genes in co-cultured $p53^{+/+}$ and $p53^{-/-}$ NPCs before and after treatment with omega-3 PUFAs EPA/DHA. Data are shown as fold change relative to untreated co-cultured $p53^{+/+}$ NPCs. Mean±SEM, n=3 independent experiments, *p<0.05, **p<0.01, ***p<0.001, Two-way ANOVA with Tukey's multiple comparisons test.

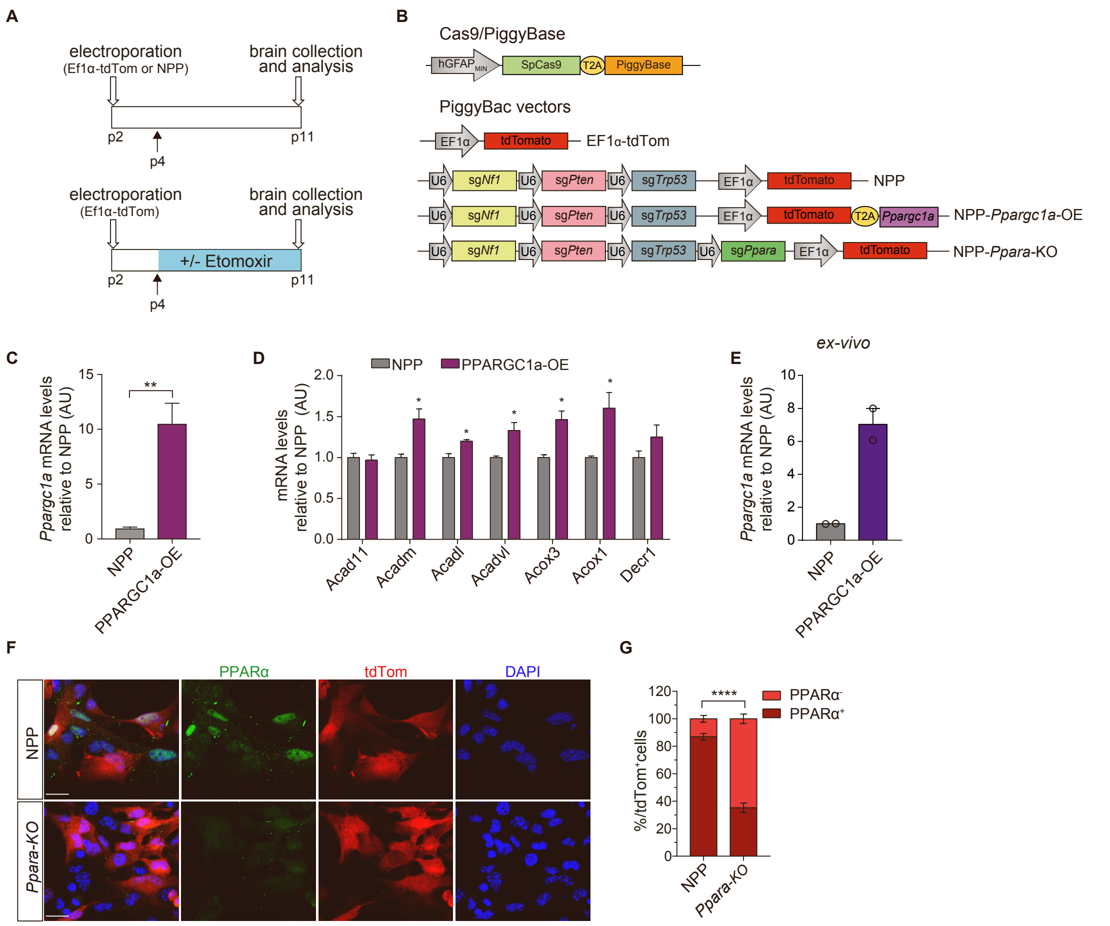

Figure S4. Validation of PPARGC1a overexpression and PPARα genetic deletion, Related to Figure 4.

A, Schematic of experimental outline**.** Pups were electroporated with the EF1α-tdTomato or NPP (*Nf1, Pten, Trp53*) plasmids at P2 and brains collected 9 days after electroporation for analysis (P11, top). Etomoxir or vehicle control were administered to EF1α-tdTomato pups from P4 every other day and brains were collected at P11 (bottom). **B**, Schematic of the Cas9/PiggyBase and PiggyBac plasmids used to generate *Nf1, Pten and Trp53* knock-out tumours (NPP), or NPP tumours overexpressing *Ppargc1a* (NPP-*Ppargc1a*-OE) or knock-out for PPARα (NPP-*Ppara*-KO). **C-D**, Validation of *Ppargc1a* overexpression by qRT-PCR analysis (C) and analysis of FAO genes expression in NPCs transfected with the NPP-*Ppargc1a*-OE plasmid (D). NPP transfected cells were used as control. Fold changes relative to NPP cells are shown. Mean±SEM, n=3 independent experiments, *p<0.05, **p<0.01, unpaired two-tailed Student t-test. Note that the majority of FAO genes are induced by PPARGC1a overexpression. **E**, qRT-PCR analysis to assess *Ppargc1a* overexpression in tdTom^{+} FACS sorted cells isolated from tumours. Mean±SEM, n=2 independent experiments. **F**, Validation of PPARα deletion by immunocytochemistry. Shown are confocal images of tdTom^{+} NPP and NPP-*Ppara*-KO cells isolated from tumours. PPARα is shown in green, transformed NPCs are tdTom^{+} (red) and nuclei are counterstained with DAPI (blue). Scale bar=20µm. **G**, Quantification of the PPARα^{+} and PPARα^{-} cells from the fluorescence images showed in F. Data are shown as percentage of the total number of tdTom^{+} cells. Mean±SEM, ****p<0.0001, unpaired two-tailed Student t-test.

Gene	Forward primer (5'-3')	Reverse primer (5'-3')
18S	CTTAGAGGGACAAGTGGCG	ACGCTGAGCCAGTCAGTGTA
Acad11	TGGCTAACATGTACGCCATCA	ATCTTGGCGATCGCTGAGA
Acadl	TCTTTTCCTCGGAGCATGACA	GACCTCTCTACTCACTTCTCCAG
Acadm	AGGGTTTAGTTTTGAGTTGACGG	CCCCGCTTTTGTCATATTCCG
Acadvl	CTACTGTGCTTCAGGGACAAC	CAAAGGACTTCGATTCTGCCC
Acox1	GCTGAGGAACCTGTGTCTCT	TCAAAGGCATCCACCAAAGC
Acox3	GCATCCTCCCAGAGTCCTAC	AGGGTGGGAGGGTAGAGATT
Ascl1	ATGCAGCTACTGTCCAAACG	AACAGTAAGGGGTGGGTGTG
Cpt1c	CGGGTTGGACAGCATTTCAA	AGCAACACCTTCCATCCTGA
DCX	AACCGGTGAGTGGGGCTTTCG	GGTGGAACCACAGCAACTTTT
Decr1	GAGCTGCGTTTCTTGCCATC	GACGACCACAGGGGATTCTG
Dll1	CTACTACGGAGAAGGTTG	GTATCCATGTTGGTCATC
Egfr	GCTTGCAACGGTTCTCTCTC	CCACTGCCATTGAACGTACCCAG
Gfap	ACCATTCCTGTACAGACTTTCTCC	AGTCTTTACCACGATGTTCCTCTT
Glast	AAGCATCACAGCCACGGCCG	GTTCGGAGGCGGTCCAGAAACC
Hes5	ATGCTCAGTCCCAAGGAGAA	TAGTCCTGGTGCAGGCTCTT
Hey1	AAAATGCTGCACACTGCAGG	CGAGTCCTTCAATGATGCTCAG
Ki67	CATGAGGATGGAAGCAAGCC	TGCTGTTCTACATGCCCTGA
Mcm2	AAGGCTGGCATCGTTACCTC	CAAAGCGGGAAATGATGGGC
NCam1	AGGGCAAGGCTGCTTTCT	CCCCATCATGGTTTGGAGT
Nestin	CTGCAGGCCACTGAAAGTT	GACCCTGCTTCTCCTGCTC
p53	GGACGGGACAGCTTTGAGGT	GTGGGCAGCGCTCTCTTTG
Pgc1a	TGATGTGAATGACTTGGATACAGAC	GCTCATTGTTGTACTGGTTGGATA
Prom1	GCCTCTACCCTGGAAGCAAA	GATGCTGGTGGATGGCTCTT
Sox2	CATGGGCTCTGTGGTCAAGT	TACATGGTCCAATTCCCCCG
Vcam	AAGAGAACCCAGGTGGAGGT	TCTGCTAATTCCAGCCTCGT

Table S2. List of quantitative RT-PCR primer sequences used in this study, Related to STAR Methods.

References

S1. Codega, P., Silva-Vargas, V., Paul, A., Maldonado-Soto, R., Angel, Deleo, M., Annina, Pastrana, E., and Doetsch, F. (2014). Prospective Identification and Purification of Quiescent Adult Neural Stem Cells from Their In Vivo Niche. Neuron *82*, 545-559. 10.1016/j.neuron.2014.02.039.

S2. Llorens-Bobadilla, E., Zhao, S., Baser, A., Saiz-Castro, G., Zwadlo, K., and Martin-Villalba, A. (2015). Single-Cell Transcriptomics Reveals a Population of Dormant Neural Stem Cells that Become Activated upon Brain Injury. Cell Stem Cell *17*, 329-340. 10.1016/j.stem.2015.07.002.

S3. Mizrak, D., Levitin, H. M., Delgado, A. C., Crotet, V., Yuan, J., Chaker, Z., Silva-Vargas, V., Sims, P. A., and Doetsch, F. (2019). Single-Cell Analysis of Regional Differences in Adult V-SVZ Neural Stem Cell Lineages. Cell Rep *26*, 394-406.e395. 10.1016/j.celrep.2018.12.044.
